# Supplementary material for: Local particle-hole pair excitations by SU(2) symmetry fluctuations
Source: Sci Rep. 2017 Jun 14;7:3477. doi: 10.1038/s41598-017-01538-1 (PMC5471275; doi:10.1038/s41598-017-01538-1)
Supplement: Supplementary file 1 — Supplementary Information [file 41598_2017_1538_MOESM1_ESM.pdf]

Local particle-hole pair excitations by SU(2) symmetry fluctuations

X. Montiel[1,2], T. Kloss[1,3], and C.Pépin[1,*]

[1]IPhT, L'Orme des Merisiers, CEA-Saclay, 91191 Gif-sur-Yvette, France
[2]Department of Physics, Royal Holloway, University of London, Egham, Surrey TW20 0EX, United Kingdom
[3]INAC-PHELIQS, Université Grenoble Alpes and CEA, 38000 Grenoble, France
[*]catherine.pepin@cea.fr

Supplementary Information

The SU(2) symmetry

The SU(2) symmetry emerges naturally from the Eight-Hot Spots (EHS) model[1,2] where the electronic dispersion has been linearized around the hot-spots (points of the Fermi surface related by the Antiferromagnetic modulation vector $\mathbf{Q} = (\pi, \pi)$). The SU(2) symmetry allows the rotation between the d-wave SC order to the d-wave charge channel. In the framework of the EHS models, the charge order modulation vector is defined as $\mathbf{Q}_0 = (\pm \mathbf{Q}_a, \pm \mathbf{Q}_b)$ and relates two hot-spots on opposite Fermi surface. In our model, we need to generalize this symmetry to the whole Fermi surface.

We define the SU(2) pseudo-spin operator $\eta^+, \eta^- = (\eta^+)^\dagger$ and η_z associated with the SU(2) symmetry as :

$$\eta^+ = \sum_{\mathbf{k}} \psi^\dagger_{\mathbf{k}\uparrow} \psi^\dagger_{-\mathbf{k}+\mathbf{Q}_0\downarrow} \tag{1}$$

$$\eta_z = \frac{1}{2} \sum_{\mathbf{k}} \left(\psi^\dagger_{\mathbf{k}\uparrow} \psi_{\mathbf{k}\uparrow} + \psi^\dagger_{-\mathbf{k}+\mathbf{Q}_0\downarrow} \psi_{-\mathbf{k}+\mathbf{Q}_0\downarrow} - 1 \right), \tag{2}$$

The operators in Eq. 1 and Eq. 2 are called pseudo-spin operators and constitute a SU(2) algebra. Here, these operators act on a $l = 1$ triplet representation involving two conjugate d-wave SC operators (Δ_{-1} and Δ_1) and a d-wave charge sector operator Δ_0 which writes :

$$\Delta_{-1} = \frac{1}{\sqrt{2}} \sum_{\mathbf{k}} d_{\mathbf{k},\mathbf{Q}_0} \psi_{\mathbf{k}\downarrow} \psi_{-\mathbf{k}\uparrow}, \tag{3}$$

$$\Delta_0 = \frac{1}{2} \sum_{\mathbf{k},\sigma} d_{\mathbf{k},\mathbf{Q}_0} \psi^\dagger_{-\mathbf{k}+\mathbf{Q}_0\sigma} \psi_{-\mathbf{k},\sigma}, \tag{4}$$

$$\Delta_1 = -\frac{1}{\sqrt{2}} \sum_{\mathbf{k}} d_{\mathbf{k},\mathbf{Q}_0} \psi^\dagger_{\mathbf{k}\uparrow} \psi^\dagger_{-\mathbf{k}\downarrow}. \tag{5}$$

The form factor is given by $d_{\mathbf{k},\mathbf{Q}_0} = \left(d_{\mathbf{k}} + d_{\mathbf{Q}_0}\right)/2$ with $d_{\mathbf{k}} = \left(cos(k_x a) + cos(k_y a)\right)/2$. The standard SU(2) relations

$$\left[\eta^\pm, \Delta_m\right] = \sqrt{l\,(l+1) - m\,(m \pm 1)}\Delta_{m\pm1} \tag{6}$$

and

$$\left[\eta_z, \Delta_m\right] = m \Delta_m, \tag{7}$$

are valid here.

Calculation of the charge density on oxygen atoms

In order to evaluate the charge on the Oxygen atoms, we calculate the charge density on the bond between two adjacent Copper atoms. The charge density at points $\mathbf{R}$ and $\mathbf{R}'$, writes :

$$\begin{aligned}
\rho\left(\mathbf{R}, \mathbf{R}'\right) &= \langle \psi^\dagger_{\mathbf{R},\sigma} \psi_{\mathbf{R}',\sigma} \rangle + h.c. \\
&= \frac{1}{N} \sum_{\mathbf{k},\mathbf{k}'} \exp\left[i \frac{\mathbf{k}+\mathbf{k}'}{2} \cdot \left(\mathbf{R} - \mathbf{R}'\right)\right] \exp\left[i \frac{\mathbf{k}-\mathbf{k}'}{2} \cdot \left(\mathbf{R} + \mathbf{R}'\right)\right] \\
&\times \left(\langle \psi^\dagger_{\mathbf{k},\sigma} \psi_{\mathbf{k}',\sigma} \rangle + h.c. \right),
\end{aligned} \tag{8}$$

where $\mathbf{R}$ is the coordinate of the Copper atoms on a square lattice with $\mathbf{a_0^x}$, $\mathbf{a_0^y}$ the unit wave vectors. The charge on the oxygen atoms are generated by perturbation theory on the three bands model and is proportional to the bond density

$$\rho_{O,i}\left(\mathbf{R}\right) \sim \rho\left(\mathbf{R}, \mathbf{R} \pm \mathbf{a_0^i}\right), \tag{9}$$

with $i = \{x, y\}$[2]. We relate now the correlation function $\left(\langle \psi^\dagger_{\mathbf{k},\sigma} \psi_{\mathbf{k}',\sigma} \rangle + h.c. \right)$ to the Green function of the RES and notice that the inter cell modulations corresponds to $\mathbf{k} - \mathbf{k}' = \mathbf{P}$, with $\mathbf{P} = \{\mathbf{2k_F}\}$. The charge density on the oxygen atoms Eqn.(9) then writes :

$$\rho_{O,i}\left(\mathbf{r}\right) = \frac{4T}{N} \sum_{\varepsilon,\mathbf{k},\mathbf{P}} \cos\left(\left(\mathbf{k} + \frac{\mathbf{P}}{\mathbf{2}}\right) \cdot \mathbf{a_0^i} \right) \cos\left(\mathbf{P} \cdot \mathbf{R}\right) G^{RES}(\varepsilon, \mathbf{k}, \mathbf{P}) \tag{10}$$

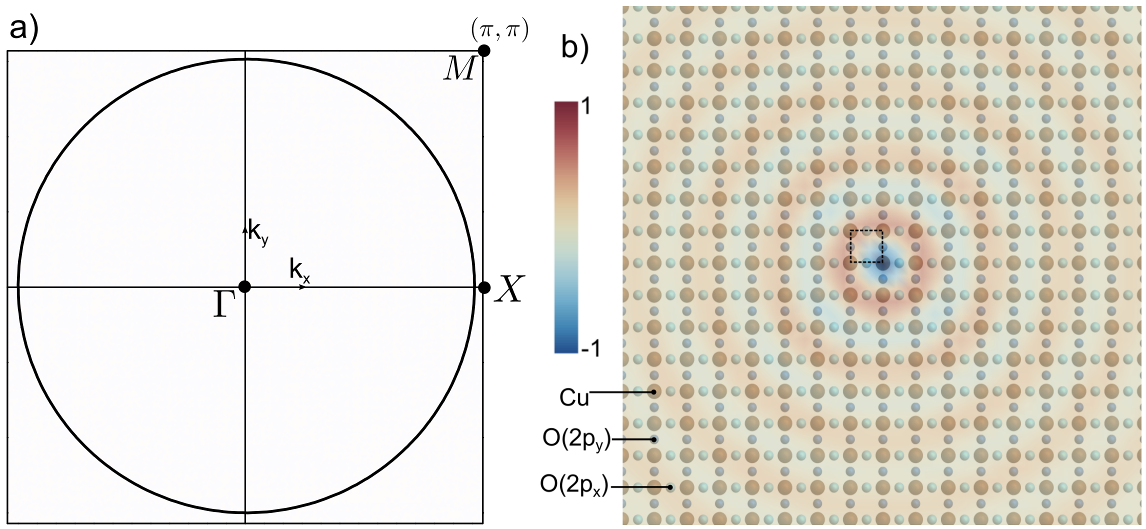

Figure 1. RES in the real space in two dimension in the case of a circular Fermi surface. In a) we present the circular Fermi surface in the first Brillouin zone. The gap is constant in the whole Brillouin zone and is magnitude is set to $50\,meV$. In b), we show the corresponding spacial dependence of the charge density on the copper atoms with a s-wave symmetry gap.

where the sum over the momentum $\mathbf{k}$ is done in the first BZ, ε is the fermionic Matsubara frequency and $G^{RES}(\varepsilon,\mathbf{k},\mathbf{P})$ is the RES Green function that writes:

$$G^{RES}(\varepsilon,\mathbf{k},\mathbf{P}) = -\frac{\chi^{\mathbf{RES}}_{\mathbf{k},\mathbf{P}}}{(\mathbf{i}\varepsilon-\xi_{\mathbf{k}+\frac{\mathbf{P}}{2}})(\mathbf{i}\varepsilon-\xi_{\mathbf{k}-\frac{\mathbf{P}}{2}})-\left(\chi^{\mathbf{PG}}_{\mathbf{k},\mathbf{P}}\right)^{\mathbf{2}}}. \tag{11}$$

We choose the tight binding fermionic dispersion ξ_k that fits the experimental dispersions of Bi2212 compound[3]. The gap $\chi^{RES}_{\mathbf{k},\mathbf{P}}$ is chosen to reproduce the solution of the mean field equation with a d-wave symmetry. For each $\mathbf{P}$, the gap develops in a small area close to the Fermi surface. Ignoring the temperature and frequency dependence of the order parameter, we perform the sum over the fermionic Matsubara frequencies analytically. The charge density on the O atoms writes:

$$\begin{aligned}\rho_O(\mathbf{r}) = \tfrac{4}{N}\textstyle\sum_{\mathbf{k},\mathbf{P}} \Big[&\cos\left(\left(\mathbf{k}+\tfrac{\mathbf{P}}{\mathbf{2}}\right)\cdot\mathbf{a^i_0}\right)\cos\left(\mathbf{P}\cdot\mathbf{R}\right)\\ &\times\tfrac{\chi^{RES}_{\mathbf{k},\mathbf{P}}\left[n_F(E^+_{\mathbf{k}})-n_F(E^-_{\mathbf{k}})\right]}{E^+_{\mathbf{k}}-E^-_{\mathbf{k}}}\Big].\end{aligned} \tag{12}$$

where $i=\{x,y\}$, n_F is the Fermi-Dirac distribution and $E^{\pm}_{\mathbf{k}} = \frac{1}{2}\left(\xi_{\mathbf{k}+\frac{\mathbf{P}}{2}}+\xi_{\mathbf{k}-\frac{\mathbf{P}}{2}}\right) \pm \frac{1}{2}\sqrt{\left(\xi_{\mathbf{k}+\frac{\mathbf{P}}{2}}-\xi_{\mathbf{k}-\frac{\mathbf{P}}{2}}\right)^2+|\chi^{RES}_{\mathbf{k},\mathbf{P}}|^2}$ are the energies of the charge excitons. The RES order parameter $\chi^{RES}_{\mathbf{k},\mathbf{P}}$ has a d-wave form factor which produces an opposite sign between the O atom charge density along the x and the y axis.

Charge density in the s-wave symmetry gap

We present below the local $2\mathbf{p}_\mathrm{F}$ solution for a circular Fermi surface subjected to s-wave pairing, which can be seen for example, in the case of the attractive Hubbard model. The local structure of the s-wave mode is reminiscent of a Friedel oscillation (see figure 1).

Evolution of the order parameter with the magnitude of the interaction $\bar{\pi}_0$

We present the effect of the magnitude of the SU(2) pairing fluctuation $\bar{\pi}_0$ (see equation (2) on the gap dependence $\chi_{\mathbf{k},\mathbf{k}'}$. We resolve the equation (8 considering a mass hich is maximal in the nodal zone and vanihes in the AN zone $\bar{a}_{0,k} = m_0 exp(-d_k^2/w)$ with $d_k = 0.5*(cos(k_x)-cos(k_y))$ and $m_0 = 100$ and $w = 1$. The coefficient $J_1 = 0.1$ does not qualitatively affect the results. We choose $\mathbf{k}' = \mathbf{k}-\mathbf{P}$ with $\mathbf{P} = 2\mathbf{p}_\mathrm{F}$. In the figure 2, we present the effect of the magnitude of the interaction $\bar{\pi}_0$. We clearly demonstrate that the size of the gapped zone increases with the magnitude of the interaction.

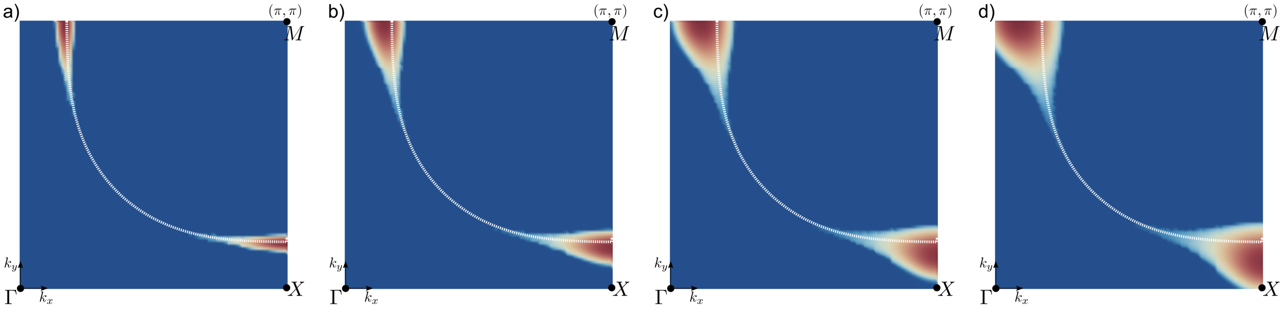

Figure 2. Effect of $\bar{\pi}_0$ on the momentum dependence of $\chi_{\mathbf{k};\mathbf{P}}$ We present the momentum dependence of the charge order parameter $\chi_{\mathbf{k},\mathbf{P}}$ in the positive quarter of the first BZ for a) $\bar{\pi}_0 = 0.25$, b) $\bar{\pi}_0 = 0.5$, c) $\bar{\pi}_0 = 0.75$ and d) $\bar{\pi}_0 = 1$. The size of the gapped zone increases with the magnitude $\bar{\pi}_0$.

References

1. Metlitski, M. A. & Sachdev, S. Quantum phase transitions of metals in two spatial dimensions. II. Spin density wave order. *Phys. Rev. B* **82**, 075128 (2010). DOI 10.1103/PhysRevB.82.075128.

2. Efetov, K. B., Meier, H. & Pépin, C. Pseudogap state near a quantum critical point. *Nat. Phys.* **9**, 442–446 (2013). DOI 10.1038/nphys2641.

3. Fujita, K. *et al.* Simultaneous Transitions in Cuprate Momentum-Space Topology and Electronic Symmetry Breaking. *Science* **344**, 612–616 (2014). DOI 10.1126/science.1248783.
